# The Identification of CD163 Expressing Phagocytic Chondrocytes in Joint Cartilage and Its Novel Scavenger Role in Cartilage Degradation

**DOI:** 10.1371/journal.pone.0053312

**Published:** 2013-01-11

**Authors:** Kai Jiao, Jing Zhang, Mian Zhang, Yuying Wei, Yaoping Wu, Zhong Ying Qiu, Jianjun He, Yunxin Cao, Jintao Hu, Han Zhu, Li-Na Niu, Xu Cao, Kun Yang, Mei-Qing Wang

**Affiliations:** 1 Department of Oral Anatomy and Physiology and TMD, School of Stomatology, Fourth Military Medical University, Xi'an, China; 2 Department of Immunology, Fourth Military Medical University, Xi'an, China; 3 Department of Orthopedics, Xijing Hospital, Fourth Military Medical University, Xi'an, China; 4 Department of Prosthodontics, School of Stomatology, Fourth Military Medical University, Xi'an, China; 5 Department of Orthopaedic Surgery, The Johns Hopkins University School of Medicine, Baltimore, Maryland, United States of America; Charite Universitätsmedizin, Germany

## Abstract

**Background:**

Cartilage degradation is a typical characteristic of arthritis. This study examined whether there was a subset of phagocytic chondrocytes that expressed the specific macrophage marker, CD163, and investigated their role in cartilage degradation.

**Methods:**

Cartilage from the knee and temporomandibular joints of Sprague-Dawley rats was harvested. Cartilage degradation was experimentally-induced in rat temporomandibular joints, using published biomechanical dental methods. The expression levels of CD163 and inflammatory factors within cartilage, and the ability of CD163^+^ chondrocytes to conduct phagocytosis were investigated. Cartilage from the knees of patients with osteoarthritis and normal cartilage from knee amputations was also investigated.

**Results:**

In the experimentally-induced degrading cartilage from temporomandibular joints, phagocytes were capable of engulfing neighboring apoptotic and necrotic cells, and the levels of CD163, TNF-α and MMPs were all increased (*P*<0.05). However, the levels of ACP-1, NO and ROS, which relate to cellular digestion capability were unchanged (*P*>0.05). CD163^+^ chondrocytes were found in the cartilage mid-zone of temporomandibular joints and knee from healthy, three-week old rats. Furthermore, an increased number of CD163^+^ chondrocytes with enhanced phagocytic activity were present in Col-II^+^ chondrocytes isolated from the degraded cartilage of temporomandibular joints in the eight-week experimental group compared with their age-matched controls. Increased number with enhanced phagocytic activity of CD163^+^ chondrocytes were also found in isolated Col-II^+^ chondrocytes stimulated with TNF-α (*P*<0.05). Mid-zone distribution of CD163^+^ cells accompanied with increased expression of CD163 and TNF-α were further confirmed in the isolated Col-II^+^ chondrocytes from the knee cartilage of human patients with osteoarthritis, in contrast to the controls (both *P*<0.05).

**Conclusions:**

An increased number of CD163^+^ chondrocytes with enhanced phagocytic activity were discovered within degraded joint cartilage, indicating a role in eliminating degraded tissues. Targeting these cells provides a new strategy for the treatment of arthritis.

## Introduction

Osteoarthritis (OA) is one of the main causes of chronic disability. Moreover, none of the therapies in current use appear to have an obvious impact on impeding or reversing the histopathological progression to advanced OA [1], mainly due to the limited understanding of its pathogenesis. Multiple catabolic factors have been investigated in the context of the breakdown of homeostasis within OA [2]. Recent studies focused on addressing the ability of chondrocytes to repair cartilage in OA, for example, by increasing matrix synthesis [3] in this avascular and alymphatic tissue [4]. At least clinically, OA can be self-limiting, with patients experiencing extended periods without further deterioration in their condition.

Prompt removal of dying cells is crucial for maintaining tissue homeostasis; phagocytosis is the key process in this regard [5]. Mature tissue macrophages form the first line of defense in recognizing and eliminating potential pathogens. The main functions of macrophages include phagocytosis and the production of inflammatory mediators, and these processes are tightly regulated by their surface receptors, which are heterogeneously expressed by mature tissue macrophages [6]. CD163, a member of the scavenger receptor cysteine-rich (SRCR) superfamily (also known as RM3/1, M130, or p155) [7], is one of the most specific surface markers for macrophages that is expressed at high levels in the majority of subpopulations of mature tissue macrophages across species [6], [8]–[11]. Tissue macrophages (for example, in liver, spleen and lymph node) show substantially higher expression of CD163 compared to monocytes [12]. The most well characterized function of CD163 relates to the internalization of the hemoglobin (Hb) - haptoglobin (Hp) complex [13]. CD163 also plays an important role in host defense, in the detection of bacterial infection [8]. Macrophages expressing increased levels of CD163 are found in inflammatory conditions [6], [14], and during wound healing [15]. The increased synthesis of CD163 can be indicative of alternative macrophage activation [16].

Chondrocytes are believed to have limited proliferative and regenerative capabilities, dependent on their location within different tissue layers [17]. In arthritic cartilage, there is an increase in the proportion of dead cells or cell debris [18]. The fate of the dead cells and cell debris is unknown, and it is unclear whether there is a role for CD163-mediated phagocytosis within cartilage. Uncovering the mechanisms responsible for removing the cellular debris by phagocytosis within degenerative tissues will facilitate an understanding of the pathogenesis of these complex diseases, such as OA and rheumatoid arthritis (RA), in which tissue homeostasis has broken down. In this study, CD163 expressing (CD163^+^) chondrocytes were identified, for the first time, in healthy knee and temporomandibular joint (TMJ) cartilage from Sprague-Dawley (SD) rats. In addition, an increased percentage of CD163^+^ chondrocytes with enhanced phagocytic activity was observed in the degraded cartilage of TMJs, which was associated with increased expression of tumor tissue necrosis factor alpha (TNF-α) [19]–[21]. Finally, increased expression of CD163 and TNF-α were confirmed in the knee cartilage from OA patients compared to healthy joints derived from amputees.

## Materials and Methods

### Sample collection

Female SD rats of three or eight weeks of age were provided by the Animal Center of the Fourth Military Medical University (Xi'an, China). The care of the animals, and all procedures were performed according to institutional guidelines, and were approved by the Ethics Committee of the Fourth Military Medical University. The rats received a standardized diet throughout the procedures, and none of the rats showed any signs of disability. In the experimental (E) groups, biomechanical dental stimulation was applied to the eight-week old female SD rats, as previously described [19]–[21]. In the sham-treated groups (control groups, C), rats underwent a mock operation procedure with no biomechanical stimulation. TMJs were harvested for morphological observations and for *ex vivo* investigations. The TMJ cartilage of three-week old rats was harvested, and the primary cells were isolated by enzyme digestion of cartilage; these cells were used for the *in vitro* experiments. Knee cartilage from patients with osteoarthritis (OA) or healthy cartilage from patients undergoing knee amputation were collected and investigated by histochemical and immunohistochemical staining and real-time PCR analysis. All patients agreed to the experimental procedures, and provided written informed consent. All procedures were approved by the Ethics Committee of the Fourth Military Medical University. Cartilage was harvested from OA patients aged 59–70 years (including three male patients, aged 53–70 years, mean age 64.3 years, and two female patients, aged 66–70 years, mean age 68 years). Healthy cartilage was harvested from patients undergoing amputations following traumatic traffic-related injuries, but in the absence of injury to the knee joint. Patients were aged 31–44 years (including four male patients aged 31–44 years, mean age 39 years and one female patient, aged 33 years). Additional details are included in the Methods S1.

### Tissue preparation for gross-, micro- and ultrastructural observations and immunohistochemistry

Using a dissecting microscope (SZX9, Olympus, Japan) six samples of the most obvious grossly damaged regions of rat TMJ cartilage were examined by transmission electron microscopy (TEM) [19]. Serial midsagittal sections (5 µm-thick) were cut from paraffin-embedded, decalcified TMJ tissue or human knee joint blocks using a microtome. Sections were stained with hematoxylin and eosin (H&E) or toluidine blue for histological assessment [19], [20]. TUNEL staining was used for the detection of dead chondrocytes. A standard, three-step, avidin-biotin complex (ABC) immunohistochemical staining protocol or indirect immunofluorescent staining protocol was carried out, as previously reported [20]. The primary antibodies were mouse anti-rat monoclonal CD163 (MCA342R, Serotec Ltd, Oxford, UK, dilution 1∶50), mouse anti-human monoclonal CD163 (SC-20066, Santa Cruz, USA, dilution 1∶50), and a goat polyclonal TNF-α antibody, which recognizes rat and human TNF-α (sc-1351, Santa Cruz, CA, USA dilution 1∶100). Negative controls were incubated with non-immune serum instead of the primary antibody. Five fields at 400× magnification were selected at random, photomicrographs were obtained and the positive cells in each image were counted. Experiments were performed in triplicate.

### Tissue preparation for real-time PCR and Western blotting

Total RNA and protein was extracted from control or experimental groups as previously described [19]. Gene expression was analyzed using the Applied Biosystems 7500 Real-Time PCR machine. The amount of target cDNA, relative to GAPDH, was calculated using the formula 2^−ΔΔCt^ [19]. For Western blots, total protein from each group (40 µg) was fractionated by SDS-PAGE and transferred onto a nitrocellulose membrane. The nitrocellulose membrane was blocked with 5% non-fat milk and incubated with the anti-CD163 (1∶200) or anti-TNF-α (1∶500) antibodies. Signals were revealed by incubation with a horseradish peroxidase-conjugated secondary antibody (1∶5000, ZhongShan Goldenbridge Biotechnology, China) and enhanced chemiluminescence detection. Additional details are included in Methods S1.

### Chondrocyte isolation

Chondrocytes were isolated from the condylar cartilage of rat TMJs by digestion with 0.25% trypsin (Sigma, St. Louis, MO, USA) for 20 min, followed by 0.2% type II collagenase (Invitrogen, San Diego, CA, USA) for 2–3 h. Cells from human knees were harvested by the same method, except that the duration of digestion with type II collagenase was increased to 9–10 h.

### Measurement of the generation of reactive oxygen species (ROS)

Intracellular ROS was detected by means of an oxidation-sensitive fluorescent probe (DCFH-DA). Chondrocytes were collected and washed twice in phosphate-buffered saline (PBS) following incubation with 10 µmol/L DCFH-DA at 37°C for 20 min according to the manufacturer's instructions (Reactive Oxygen Species Assay Kit, Beyotime Institute of Biotechnology, China). DCFH-DA was deacetylated intracellularly by a non-specific esterase, and this product was further oxidized by ROS to the fluorescent compound 2,7-dichlorofluorescein (DCF). DCF fluorescence was detected using a FACSAria flow cytometer (BD Biosciences, San Jose, CA, USA). Thirty thousand events were collected for each sample [22].

### Measurement of intracellular nitric oxide (NO) concentration

Chondrocytes were isolated from TMJ condylar cartilage for the measurement of the intracellular levels of NO using the Griess assay according to the protocol of the manufacturer (Total Nitric Oxide Assay Kit, Beyotime Institute of Biotechnology, China) [23].

### Collagen-II expressing (Col-II^+^) cell sorting

Isolated cells from cartilage were incubated at 4°C in 0.1% BSA in PBS for 40 min, incubated with biotin-conjugated Col-II antibody (1 µg/10^6^ cells, ab79127, Abcam, UK) at 4°C for 1 h, and washed twice with Dulbecco's PBS (DPBS) containing 5% FBS. Subsequently, cells were incubated with an APC-conjugated secondary antibody (1 µg/10^6^ cells, Invitrogen) for 40 min. After thorough washing, cells were resuspended in 0.5 ml DPBS and processed using a FACSAria flow cytometer (BD Biosciences). The sorted primary Col-II^+^ cells were then used for the testing of the phagocyitc function of CD163^+^ chondrocytes.

### Magnetic sorting of CD163^+^ cells

CD163 positive (CD163^+^) cells were selected by the combined use of a mouse anti-rat CD163 primary antibody (1 µg/10^6^ cells) and monosized magnetic polystyrene beads (25 µl/1×10^7^ cells) pre-coated with human anti-mouse IgG according to the manufacturer's instructions (Dynal 115.31D, Invitrogen). The sorted CD163^+^ cells were co-cultured with cell debris and were used for the observation of the phagocytosis by living cell workstation. Additional details are described in Methods S1.

### Generation of DiO-labeled cell debris and phagocytosis assay

The harvested chondrocyte from rat TMJ cartilage were resuspended at a density of 1×10^6^ cells/ml in serum-free Dulbecco's Modified Eagle's medium (DMEM). Then, 5 µl DiO solution (V-22886, Molecular Probes, Inc., USA) was added to 1 ml cell suspension and mixed well by gentle pipetting. After incubation at 37°C for 20 min, the mixture was centrifuged at 1500 rpm for 5 min and then washed twice with warm DMEM. Cell pellets were resuspended in a small amount of media, and frozen at −70°C for 20 min then thawed at 37°C for a further 20 min for ten cycles to yield the DiO-labeled cell debris.

For the phagocytosis assay, the primary Col-II^+^ cells, at a density of 1×10^6^ cells/well, were pre-incubated in DMEM containing 10% fetal bovine serum at 37°C for 48 h, then the cell debris (0.1 ml/well) was added to the wells. The mixture was incubated at 37°C, 5% CO_2_ for 48 h in DMEM supplemented with 1% FBS. The rate of phagocytosis of the cell debris was analyzed using the FITC filter of the flow cytometer.

### Exogenous TNF-α stimulation

The primary chondrocytes isolated from TMJ cartilage from three-week old SD rats were stimulated for 48 h with vehicle, 10 ng/ml TNF-α alone, or 10 ng/ml TNF-α plus 1 µg/ml CD163 neutralizing antibody (MCA342R, Serotec Ltd.). Following treatment, the cells were harvested for analysis by real-time PCR, flow cytometry and confocal microscopy.

### Flow cytometric analysis

Flow cytometry was used to detect the surface expression levels of CD163 and phagocytosis by the Col-II^+^ cells. Briefly, Col-II^+^ cells co-cultured with DiO-labeled cartilage debris were incubated at 4°C in 0.1% BSA in PBS, and then incubated with PE-conjugated CD163 antibody (1 µg/10^6^ cells, MCA342PE, Serotec Ltd.) at 4°C for 1 h. After washing, the cells were resuspended in 0.5 ml DPBS and analyzed on the flow cytometer.

### Confocal microscopy

Cells from joint cartilage that had been co-cultured with DiO-labeled cell debris were fixed with 4% formaldehyde, and incubated overnight at 4°C with the CD163 antibody (MCA342R, Serotec Ltd.). The mixture was then incubated with Cy3-conjugated antibody (1∶100, Molecular Probes, Breda, Netherlands) for 1 h, and subsequently with DAPI for 3 min at room temperature. Samples were examined using the green (blue excitation filter, 418 nm), red (green excitation filter, 514 nm) and blue (ultraviolet excitation filter, 418 nm) lasers of the confocal microscope (FV1000, Olympus, Japan). In each field of view, 10 to 15 serial optical z-axis sections (1 µm-thick) were collected using the tri-channel imaging system. Five fields of view at 400× magnification were selected at random, and the total number of CD163^+^ in each field was counted. In addition, the number of CD163^+^ cells with FITC-labeled cell debris inside their cell membrane was confirmed by the z-axis scanning (1 µm thick); these were designated phagocytic cells. Experiments were performed in triplicate.

### Living cells workstation

The sorted CD163^+^ chondrocytes were incubated with the DiO-labeled cartilage debris in DMEM at 37°C, 5% CO_2_. The living cells workstation recorded a series of images illustrating that the cell debris was undergoing phagocytosis by the CD163^+^ cells.

### Transwell migration assays

An *in vitro* migration assay was performed in 24-well transwell units (Millipore, Merck KGaA, Darmstadt, Germany) with polycarbonate filters (pore size, 8 µm), which were coated on both sides with fibronectin (3 ng/ml, Sigma) [24]. Additional details are described in Methods S1.

### Statistical analysis

Statistical analysis was performed using SPSS software, version 11.0 (SPSS, Chicago, IL, USA). All data acquisition and analysis was performed blindly. Quantitative data for control and experimental groups were subjected to one-way ANOVA and Student-Newman-Keuls (SNK-q) post-test. *P*-values of <0.05 were considered to be statistically significant.

## Results

### Increased number of CD163^+^ chondrocytes with enhanced phagocytic activity in experimentally-induced, degraded TMJ cartilage

**Figure 1:**
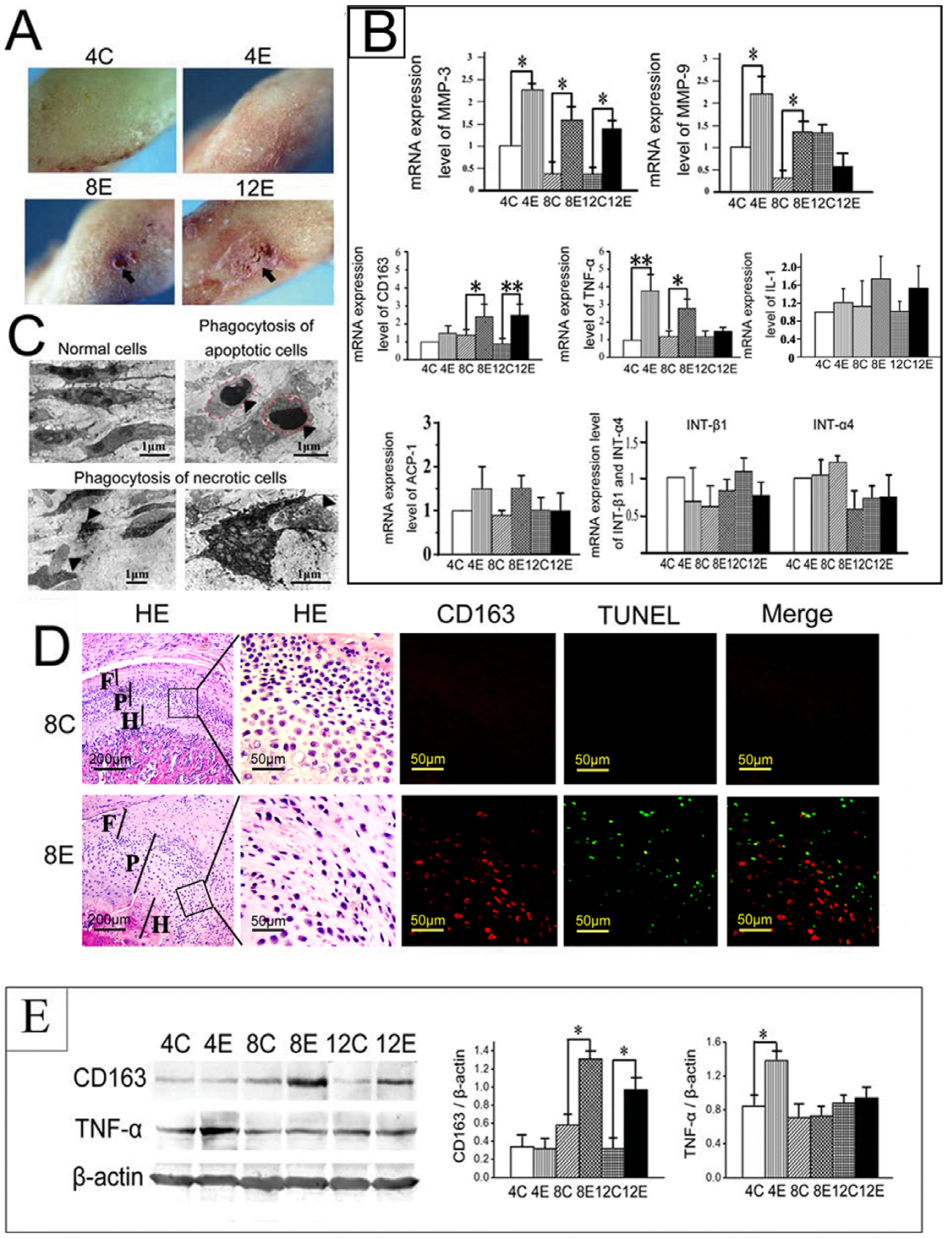
Enhanced phagocytic activity and increased CD163 and TNF-α expression in degraded TMJ cartilage. A: The gross surface morphology of rat temporomandibular joint (TMJ) condyles from control (4C) and experimental (4E, 8E, 12E) groups. Pit lesions are indicated by arrows. B: Comparison of the mRNA levels of MMP-3, MMP-9, CD163, TNF-α, IL-1, ACP-1, integrin-β1 and integrin-α4 in the condylar cartilage of control (C) and experimental (E) groups. C: Transmission electron micrographs of TMJ cartilage from control group (left top panel) and the regions with grossly damaged cartilage from experimental groups (the others panels). The apoptotic (outlined with the red dashed line) and necrotic chondrocytes are shown by arrow heads. Note that within the degraded TMJ cartilage some cells were phagocytizing neighboring apoptotic and necrotic cells. D: Serial sections of condylar cartilage from the 8-week old control (upper panels) and experimental (lower panels) groups, stained with H&E (HE), or co-stained with CD163 and TUNEL. F: fibrous layer; P: proliferative layer; H: hypertrophic layer. E: Comparison of the protein levels of CD163 and TNF-α in the condylar cartilage of control (C) and experimental (E) groups by Western blotting (left panel). Graph representing the quantification of the Western blotting results, normalized to the expression of β-actin. *P<0.05, **P<0.01. 4C: 4-week old control group; 4E: 4-week old experimental group; 8C: 8-week old control group; 8E: 8-week old experimental group; 12C: 12-week old control group; 12E: 12-week old experimental group.

The degradation of TMJ cartilage was induced by our recently reported biomechanical dental stimulation method [19]–[21]. The induced lesions within the TMJ condyles included dark, unsmooth cartilage surfaces in the 4-week old experimental group and obvious pit lesions in the 8- and 12-week old experimental groups (Figure 1A, arrow). The histological appearance of degraded cartilage, as previously reported [19], [20], included fibrillation, and condensed, eosinophilic nuclei, which was accompanied by significantly increased mRNA levels of MMP-3 and -9 (Figure 1B, *P*<0.05) [21]. To explore whether inflammation was involved in the pathogenesis of cartilage degradation, the expression of inflammatory cytokines was investigated. The results showed that increased mRNA expression of TNF-α, but not IL-1, was observed in the experimental groups compared with their age-matched controls (Figure 1B). Since chondrocytes are the only cell type within cartilage and as they remain within various stages of differentiation, we speculated that chondrocytes may take on the role of inflammatory cells within the joint cartilage. In support of this, we found apoptotic and necrotic cells within the mid-zone of degraded TMJ cartilage (Figure 1C, arrowheads), and several of these cells were being engulfed by phagocytic chondrocytes (Figure 1C). The CD163^+^ cells were located close to the TUNEL-positive dead cells in the mid-zone of the degraded cartilage in the 8-week experimental group, but not in the age-matched controls (Figure 1D). In addition, a significant increase in the mRNA and protein levels of CD163 was found in the 8- and 12-week experimental groups, compared to their age-matched controls. The increase in the expression of TNF-α was already apparent within the 4-week experimental group (Figures 1B and 1E; *P*<0.05).

**Figure 2:**
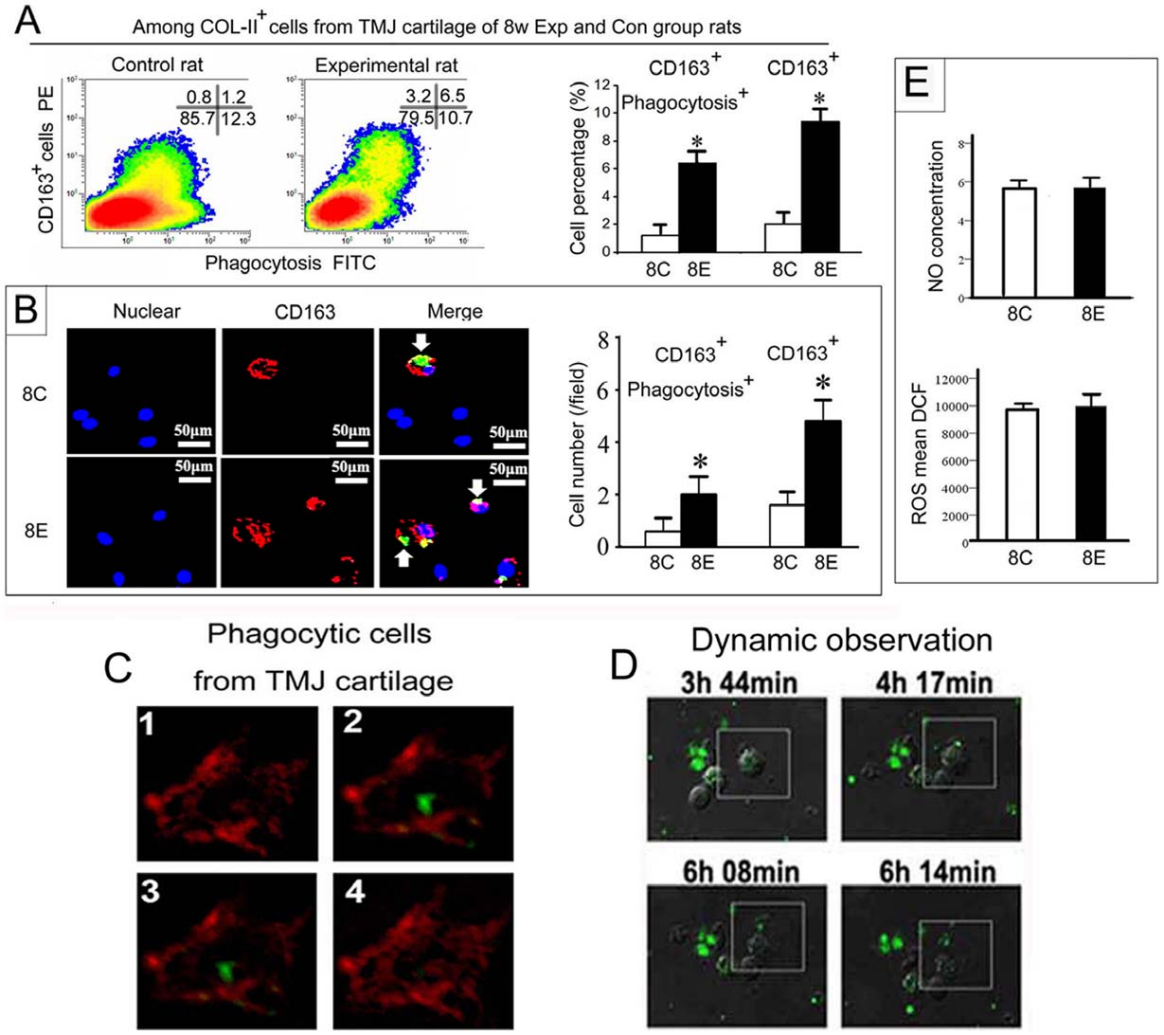
Increase in CD163^+^ cells with enhanced phagocytic activity in experimentally-induced arthritic cartilage of rat TMJs. A: Flow cytometry analysis and comparison of the percentage of total CD163^+^ cells and CD163^+^ cells with phagocytic activity within isolated type II collagen expressing (Col-II^+^) cells from TMJ cartilage from the 8-week experimental group and their age-matched controls. B: Confocal microscope images of the CD163^+^ cells and assessment of their phagocytic activity in primary cells isolated from TMJ cartilage. The images reveal an increase in CD163^+^ cells and enhanced co-localization with the FITC-labeled cell debris in 8-week experimental group compared with the age-matched controls. C: Serial confocal images (1–4) of the primary cells isolated from TMJ cartilage of 3-week old rats co-cultured with DiO-labeled cellular debris. Sections were stained with a CD163 antibody and a Cy3-conjugated secondary antibody. Note that the CD163^+^ cells showed membrane staining (red) and the cell debris (green) was located inside the cell membrane. D: Dynamic observation of the phagocytic process involving living CD163^+^ cells sorted from TMJ cartilage engulfing cellular debris. Note that the DiO-labeled debris is undergoing phagocytosis by the CD163^+^ cell indicated within the white box. E: Comparison of the nitric oxide (NO) concentration and amount of intracellular reactive oxygen species (ROS) in the primary cells isolated from TMJ cartilage from 8-week experimental group and their age-matched controls. *P<0.05.

To confirm the chondrocytic origin of this subset of CD163^+^ phagocytes in cartilage, type II collagen-expressing (Col-II^+^) chondrocytes were isolated from TMJ cartilage of 8-week experimental and control groups. The CD163^+^ cells constituted approximately 2.2% of the Col-II^+^ chondrocytes sorted from condylar cartilage of 8-wk control rats. However, the number of CD163^+^ cells and their phagocytic activity were significantly higher in the experimental group compared with the age-matched control group (Figure 2A; *P*<0.05). This result was verified by confocal microscopy, where it was observed that the number of CD163^+^ chondrocytes significantly increased in TMJ cartilage of rats in the 8-week experimental group (Figure 2B; *P*<0.05), irrespective of whether they co-localized with the cell debris. The phagocytic activity of CD163^+^ chondrocyte**s** was verified by examining serial z-sections, which showed that the cellular debris was located inside the CD163^+^ cells isolated from TMJ cartilage (Figure 2C). Moreover, this was confirmed by dynamic confocal microscopy showing the DiO-labeled cellular debris undergoing phagocytosis by the sorted CD163^+^ chondrocytes (Figure 2D; white frame). However, the ability of these phagocytic cells to digest the cellular debris appears limited because no increase in the amount of ROS or nitric oxide (NO) was detected (Figure 2E). In addition, there was no increase in mRNA expression of ACP-1, integrin β1 or integrin α4 (Figure 1B), molecules that play roles in cellular digestion and adhesion, respectively, in isolated chondrocytes from experimental groups compared with their age-matched controls (*P*<0.05).

**Figure 3:**
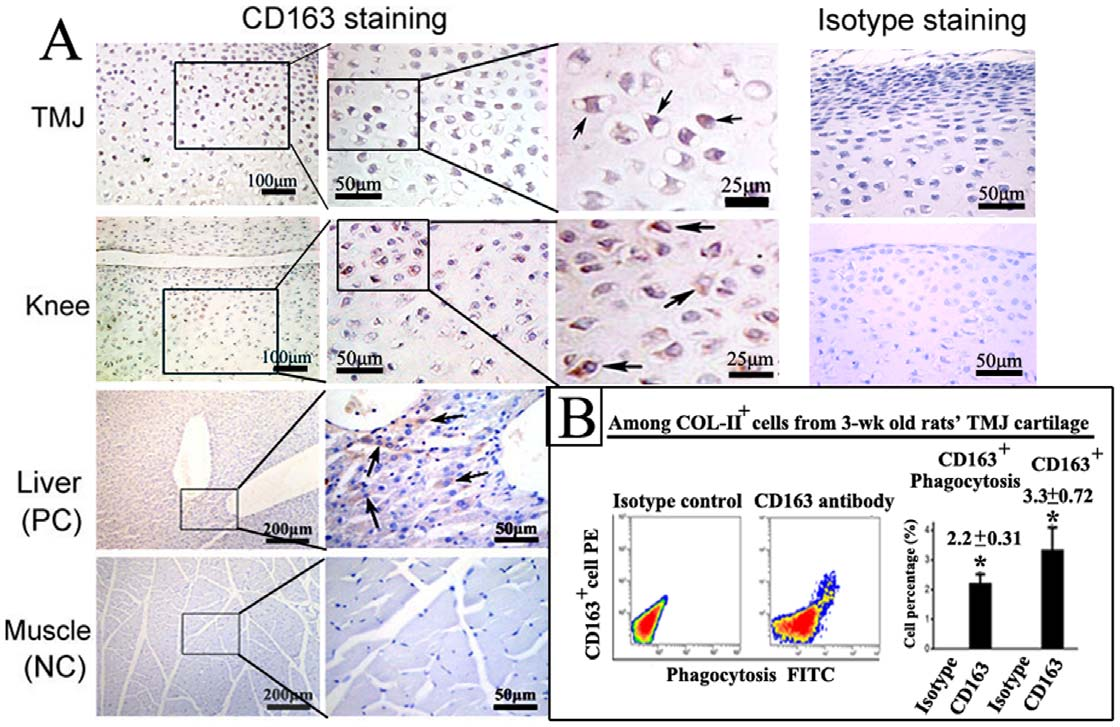
CD163^+^ chondrocytes in normal joint cartilage of 3-week old rats. A: Immunohistochemical staining of CD163 in cartilage from the TMJ and knee. The CD163^+^ cells located below the superior zone of the TMJ and knee cartilage, show intense membrane and cytoplasmic staining (arrows). Rat liver and muscle were selected as positive and negative controls, respectively, for the detection of CD163. Membrane staining of CD163^+^ cells was observed in liver (indicated by arrows), but no CD163^+^ cells were detected in muscle. As additional controls, TMJ and knee cartilage was also stained with an isotype control antibody. B: Flow cytometric analysis and graphical representation of the percentage of total CD163^+^ cells and CD163^+^ cells with phagocytic activity within the Col-II^+^ cells isolated from TMJ cartilage (n = 3; *P<0.05).

In addition, immunohistochemical staining showed that CD163^+^ cells were located in the mid-zone of cartilage in the knees and TMJs of the 3-week old healthy rats (Figure 3A). The CD163^+^ cells constituted approximately 3.3% of the Col-II^+^ cells isolated from TMJ cartilage of 3-week old healthy rats, and approximately 70% of these cells possessed phagocytic activity (Figure 3B).

Taken together, these results indicate the potential capability of the joint cartilage to actively eliminate the degraded tissues by increasing the number of CD163^+^ chondrocytes and their phagocytic activity.

### Exogenous TNF-α increased CD163 expression in primary chondrocytes and promoted migration and phagocytosis

**Figure 4:**
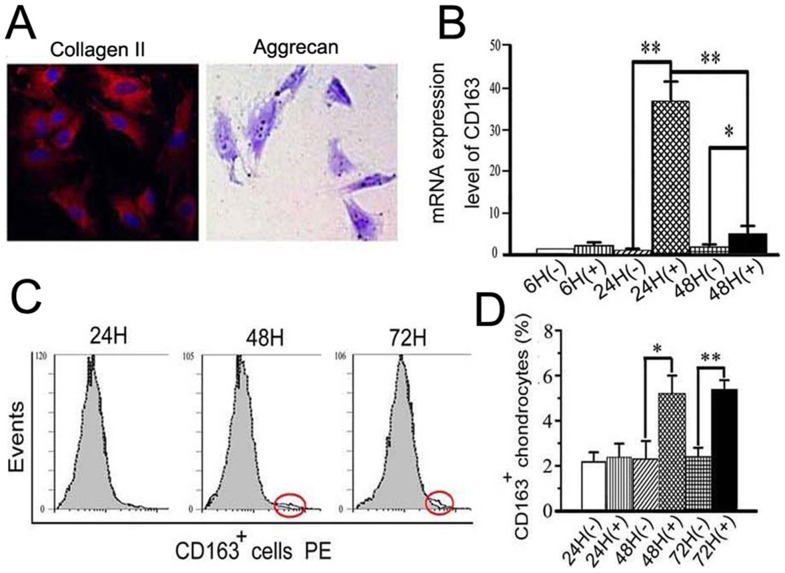
Exogenous TNF-α increased CD163 expression in primary chondrocytes from TMJ cartilage of 3-week old rats. A: The primary cells isolated from TMJ cartilage of 3-week old rats were positive for type II collagen (Col-II) and aggrecan, as detected by immunofluorescence and toluidine blue, respectively (400× magnification). B: A time-course of induction of CD163 mRNA expression in primary cells isolated from TMJ cartilage and treated with 10 ng/ml of TNF-α. C–D: Flow cytometric analysis and graphical representation of the percentage of CD163^+^ cells within the primary cells isolated from TMJ cartilage and treated with 10 ng/ml of TNF-α.

**Figure 5:**
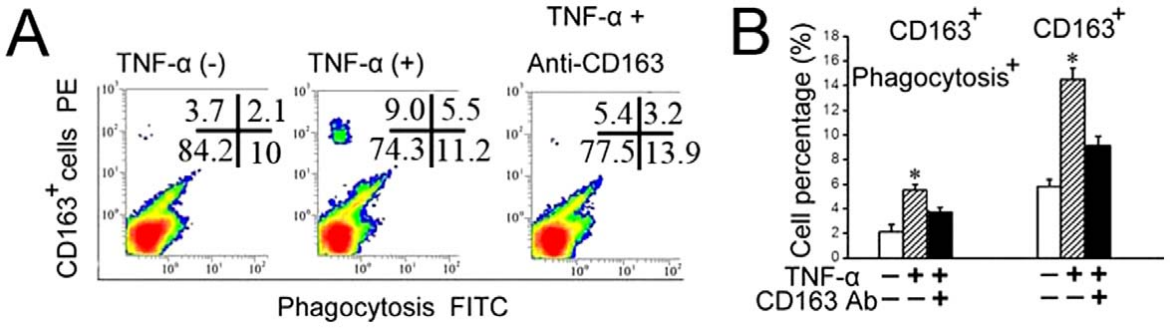
TNF-α increased the phagocytic activity of CD163^+^ cells isolated from 3 week old rat TMJ cartilage. A–B: Flow cytometry analysis (A) and graphical representation (B) of the percentage of total CD163^+^ cells and CD163^+^ cell with phagocytic activity within the primary cells isolated from TMJ cartilage and treated with vehicle, TNF-α alone, or TNF-α and a CD163 neutralizing antibody.

**Figure 6:**
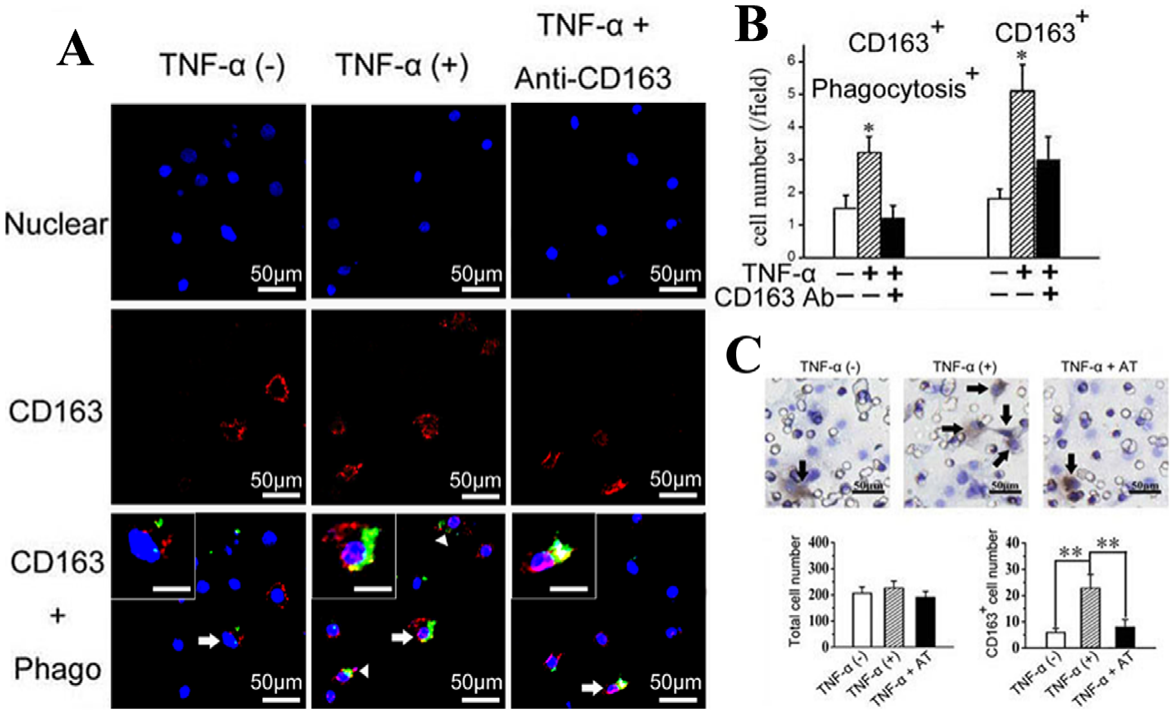
TNF-α increased the phagocytic and migratory activities of CD163^+^ cells isolated from rat TMJ cartilage. A–B: Confocal microscope images (A) and graphical representation (B) of the numbers of CD163^+^ cells and their phagocytic activity within primary cells isolated from TMJ cartilage and treated with vehicle, TNF-α alone, or TNF-α and a CD163 neutralizing antibody. The co-localization of the CD163^+^ cell with DiO-labeled cell debris (arrows), indicates that the cell debris is undergoing phagocytosis by the CD163^+^ cells, as shown in the insets. Bar: 50 µm. C: Transwell assay combined with immunohistochemical staining of CD163 indicates the migratory potential of CD163^+^ cells in response to 10 ng/ml TNF-α, which is impaired in the presence of the TNF-α neutralizing antibody (AT, 1 µg/ml). Arrows indicate the migrating CD163^+^ cells. Five fields were selected at random (at 200× magnification), and the number of CD163^+^ cells and total cells in each image were counted. **P<0.01.

TNF-α is believed to be a critical mediator in the disturbed metabolism and enhanced catabolism of degraded joint cartilage, even in the early stages of cartilage degradation [25]. In chondrocytes, TNF-α alters the expression of many molecules that contribute to cartilage degradation [26]. The results presented here indicate that the expression of TNF-α was increased at the very earliest stages of cartilage degradation, that is, only four weeks after biomechanical dental stimulation. Therefore, we wanted to address whether the increased number of CD163^+^ chondrocytes and their enhanced phagocytic activity within the degraded cartilage were attributable, at least in part, to the increase in TNF-α. This hypothesis was evaluated in the following *in vitro* studies. Primary chondrocytes, detected as Col-II- and proteoglycan-expressing cells (Figure 4A), were isolated from the TMJ cartilage of three-week old rats, and stimulated by exogenous TNF-α. The primary chondrocytes showed increased mRNA expression of CD163 after 24 and 48 h of TNF-α treatment (Figure 4B; *P*<0.05), and increased percentages of CD163^+^ cells were observed after 48 h and 72 h of TNF-α treatment (Figures 4C and D; *P*<0.05). In addition, the phagocytic activity of the CD163^+^ chondrocytes was significantly higher in the TNF-α treatment group compared with the controls (*P*<0.05). The number of phagocytic CD163^+^ chondrocytes remained at control levels when TNF-α was added in the presence of CD163 neutralizing antibodies (Figures 5A and B; *P*>0.05). The increased number of CD163^+^ chondrocytes with enhanced phagocytic activity was confirmed by confocal microscopy, and in some cases, the cells co-localized with cellular debris following TNF-α stimulation (*P*<0.05). Once again, this effect could be attenuated to control levels by treatment with a CD163 neutralizing antibody (Figure 6A, arrows and Figure 6B; *P*>0.05). The ability of TNF-α to enhance the phagocytic activity of CD163^+^ cells was additionally supported by the finding that CD163^+^ chondrocytes treated with exogenous TNF-α for 24 h showed enhanced migration, which could be attenuated to the level of the control by a TNF-α antibody (Figure 6C).

Collectively, these results demonstrate that there are CD163^+^ phagocytic chondrocytes in the joint cartilage. Exogenous TNF-α stimulation increased CD163 expression by the primary chondrocytes, and promoted the phagocytic and migratory activities of CD163^+^ chondrocytes.

### Knee cartilage from patients with osteoarthritis showed higher expression of CD163 and TNF-α

**Figure 7:**
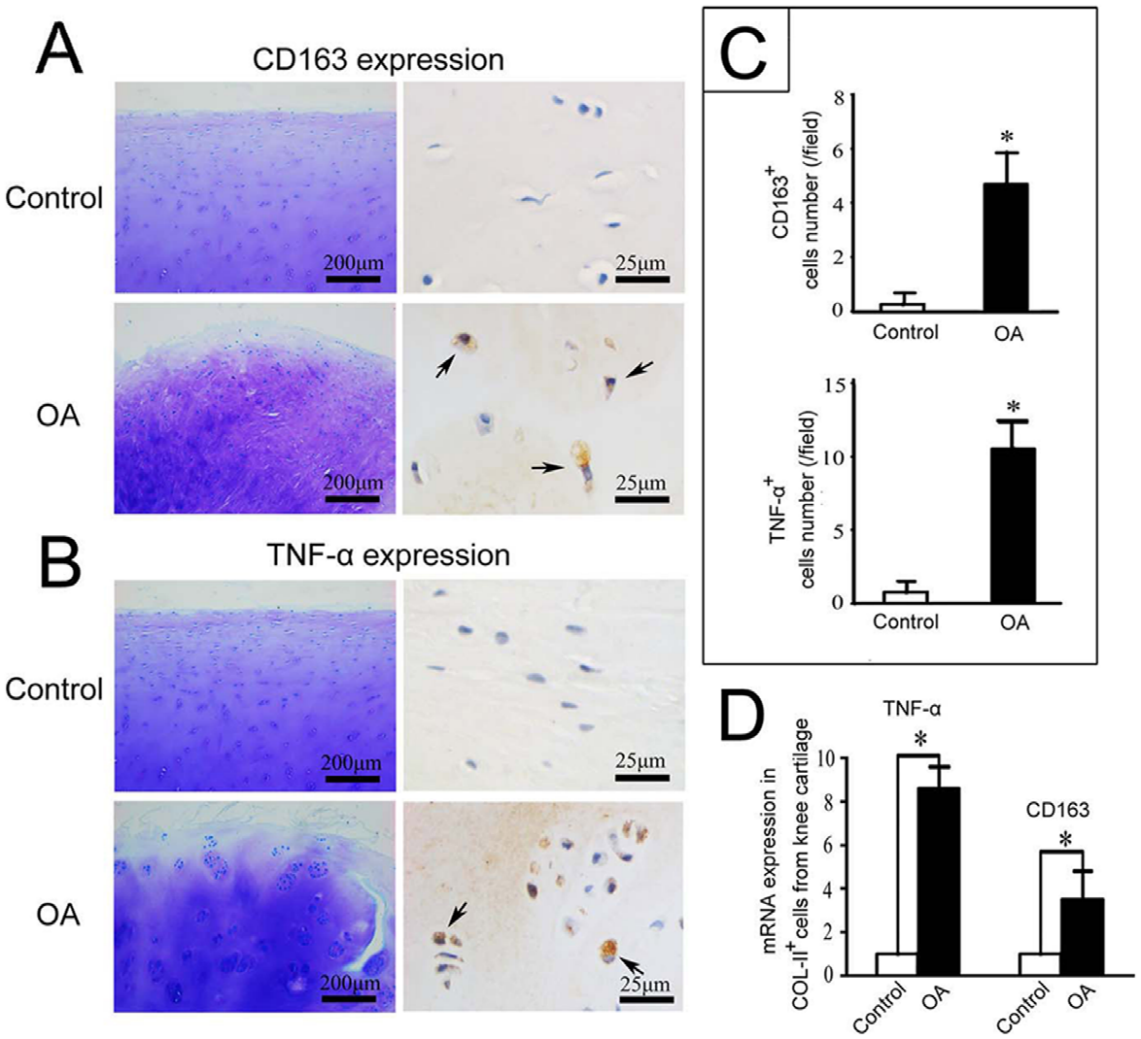
Increased expression of CD163 and TNF-α in knee cartilage from osteoarthritis patients. A and B: Toluidine blue and immunohistochemical staining of CD163 and TNF-α. C: Quantification of CD163^+^ and TNF-α^+^ cells from immunohistochemistry samples comparing the knee cartilage from patients with osteoarthritis (OA) or amputees (control). D: Comparison of the mRNA levels of TNF-α and CD163 in Col-II^+^ cells isolated from knee cartilage from OA patients or amputees (control). *P<0.05.

CD163 or TNF-α expressing cells were rarely found in amputated, healthy knee cartilage (Figures 7A and B). In contrast, significantly increased numbers of CD163^+^ and TNF-α^+^ cells were observed in the superior mid-zone of knee cartilage from patients with osteoarthritis (OA) (Figures 7A–C; *P*<0.05). In addition, the mRNA expression levels of CD163 and TNF-α were much higher in Col-II^+^ chondrocytes isolated from knee cartilage from patients with OA compared with amputees (Figure 7D; *P*<0.05).

## Discussion

The current study identified for the first time, a subset of chondrocytes within joint cartilage, characterized as CD163^+^ phagocytic chondrocytes, which are located at the mid-region, where chondrocytes are generally less differentiated. Osteoarthritis (OA)-like lesions were induced in TMJ cartilage using our previously reported biomechanical dental method [19]–[21]. Increased numbers of TNF-α expressing chondrocytes, which are usually found in degraded cartilage, were observed in these lesions [25], [26], [27]. In addition, within the OA-like cartilage there were apoptotic and necrotic cells, and an increased percentage of CD163^+^ chondrocytes with enhanced phagocytic and migratory activities. However, the scavenger function of CD163^+^ phagocytes within cartilage seems limited due to the restrictions imposed by the dense network of collagen fibrils and proteoglycans that make up articular cartilage. Degradation of the extracellular matrix by an increase in matrix metalloproteinases (MMPs), which is characteristically observed in arthritic cartilage [28]–[30], could potentially facilitate the mobilization of the CD163^+^ phagocytes.

TNF-α has been reported to alter the expression of many molecules in chondrocytes that may contribute to the degradation of cartilage [31]. The current results showed that TNF-α treatment increased CD163 expression in chondrocytes and promoted phagocytosis and migration of CD163^+^ chondrocytes. These studies indicate a novel function for TNF-α within cartilage, which is to stimulate the self-clearing potential of joint cartilage. The increased expression of CD163 was closely correlated with the enhanced phagocytosis observed within the degraded cartilage. Moreover, blocking CD163 expression using neutralizing antibodies largely attenuated the increased phagocytosis of CD163^+^ chondrocytes stimulated by TNF-α. This indicates that CD163, which is expressed in a subset of chondrocytes, may adopt the role of a scavenger receptor in order to clear the degraded tissue and maintain cartilage homeostasis. This hypothesis is supported by previous studies showing that CD163 acts as an endocytic receptor for both the hemoglobin-haptoglobin complexes and bacteria [8], [13]. However, further studies are needed to clarify the function of CD163 expressed on the phagocytic chondrocytes. Future experiments could involve the overexpression of CD163 in chondrocytes and a comparison of the difference in phagocytic potential between CD163^+^ and CD163^−^ chondrocytes.

In addition, the CD163^+^ cells constituted approximately 3.3% of the Col-II^+^ chondrocytes isolated from TMJ condylar cartilage, with approximately 70% of the cells possessing the phagocytic activity (Figure 3B). This result suggests that chondrocytes possess an inherent phagocytic/scavenger-like phenotype, which might be a general mechanism for clearing tissue debris arising from different processes within the articular cartilage, such as cartilage development and remodeling, endochondral ossification, and cartilage degradation. In the 8-week control group, the CD163^+^ cells constituted approximately 2.2% of the Col-II^+^ chondrocytes isolated from condylar cartilage (Figure 2A). This low level expression of CD163 in normal TMJ condylar cartilage may explain why the immunohistological staining was absent in the 8-week old group (Figure 1D).

The destruction of the extracellular microenvironment (ECM) facilitates the mobilization of CD163^+^ phagocytic chondrocytes. However, at the same time, this process destroys the environment that maintains the viability of the chondrocytes. This could explain the limited capacity of the CD163^+^ phagocytes to digest cellular debris, although it must be noted that the mRNA analysis was based on the analysis of all cells in the joint cartilage because the limited number of CD163^+^ cells within cartilage precluded the functional analysis of this specific cellular subset. The paradox is obvious: there is a requirement for degradation of the ECM to facilitate the mobilization of CD163^+^ phagocytes. However, ECM is needed to maintain phagocyte viability. The increased phagocytosis but limited digestion capability of this cell population within degraded cartilage may sensitize them to cell death leading to the secretion of additional inflammatory cytokines, and resulting in the progressive degradation of cartilage in arthritis.

One previous *in vitro* study using flow cytometry showed that approximately 90% of chondrocytes could phagocytose FITC-latex particles [32]. However, our pilot study performing the same experiments showed that the FITC-latex particles stick easily to the surfaces of the chondrocytes, causing false positive results (data not shown). Therefore, in the present study, DiO-labeled cell debris was used to evaluate phagocytosis. In order to exclude false results caused by non-specific adhesion, the cells were thoroughly washed prior to analysis by flow cytometry. In addition, the confocal serial z-section scans together with the images from the living cells workstation verified the phagocytic activity of CD163^+^ chondrocytes. Owing to these efforts, we have successfully identified an increase in the phagocytic activity of CD163^+^ chondrocytes from degraded cartilage of 8-week old experimental rats compared with controls, as well as in chondrocytes stimulated by TNF-α. Notably, in the present study, the percentage of CD163 negative phagocytic chondrocytes was consistently maintained at about 10% (Figure 2 and Figure 5A), irrespective of any treatment, suggesting that this phagocytic cell population within cartilage may not be as responsive to abnormal stimuli as the CD163^+^ phagocytic chondrocytes.

Increased expression of TNF-α and CD163 was observed in cartilage from OA patients compared with healthy cartilage, providing evidence that chondrocytes might undergo transdifferentiation to adopt a scavenger role. However, the gender and age difference between the two study groups should also be taken into consideration. Further clinical studies to clarify the observed difference are therefore needed, within individuals of the same gender and across a similar age distribution.

In summary, we have identified a new subset of chondrocytes, the CD163^+^ phagocytes, in joint cartilage. The results presented in this study provide new insights into the function of the chondrocytes, namely the scavenger function of CD163^+^ phagocytic chondrocytes in joint cartilage. During the early stages of cartilage degradation, some phagocytic chondrocytes appear to be capable of migrating to and clearing the degraded tissue, and therefore may have the potential to prevent further tissue damage. However, in the presence of continued stimulation, this scavenger capability would be overridden and the disease would progress. The dual role of cartilage ECM, providing cellular nutrition whilst restricting the mobilization of the defensive cartilage-resident phagocytes, offers insights for the management of OA. The therapeutic approach would require the effective elimination of the damaged tissue without extensive matrix degradation in order to provide a nutritional environment for the functional phagocytes in cartilage. Therefore, one future therapeutic strategy for arthritis could be to degrade the extracellular matrix at the early stages of the disease whilst providing cellular nutrition in homogenate form to the cartilage.

## Supporting Information

Methods S1 Supplemental material and methods.
(DOCX)
