# Supplementary material for: The Identification of CD163 Expressing Phagocytic Chondrocytes in Joint Cartilage and Its Novel Scavenger Role in Cartilage Degradation
Source: PLoS One. 2013 Jan 11;8(1):e53312. doi: 10.1371/journal.pone.0053312 (PMC3543453; doi:10.1371/journal.pone.0053312)
Supplement: Methods S1 — Supplemental material and methods. (DOCX) [file pone.0053312.s001.docx]

**Supplemental material and methods**

**Animal arrangement**

Totally one hundred and thirty female SD rats 8 wks of age were provided by the animal center of the Fourth Military Medical University. Experimental and sham control rats were respectively sacrificed at the end of the 4th, 8th or 12th week after the beginning of the experiment. For 6 rats in each time-point group, the right condyles were observed under a dissecting microscope, and then the cartilages were dissected and observed by transmission electron microscopy (TEM)^1^. The left TMJ tissue blocks were decalcified and embedded in paraffin wax for HE, TUNEL and immunohistochemical staining of CD163 and TNF-*α*^2^. For 9 rats in each time-point group, the total cartilage from the left condyles were dissected and used for real-time polymerase chain reaction (RT-PCR) analysis^1^, whereas those from the right condyles were used for western Blotting analysis^3^. For 10 rats in both 8-wk EXP group and its age-matched controls, the Collagen-Ⅱ expressing (COL-Ⅱ^+^) cells were sorted out from cells isolated from the TMJ cartilage, and were analyze by CD163 expression and phagocytic activity using flow cytometry. For the 10 rats in both 8-wk EXP group and its age-matched controls, the cells isolated from TMJ cartilage were used in determination of intracellular ROS generation and measurement of NO concentration. Knee osteoarthritis (OA) and normal (control) cartilage were provided by the orthopedic department of the Fourth Military Medical University. For 3 cartilage samples in each group, tissue blocks were decalcified and embedded in paraffin wax for toluidin blue and immunohistochemical staining of CD163 and TNF-α. For 3 samples in each group, the Collagen-Ⅱ expressing (COL-Ⅱ^+^) cells were sorted out from cells isolated from the cartilage, and were used for RT-PCR analysis of CD163 and TNF-α.

**RNA extraction and real-time PCR**

For in vivo experiment, every 3 out of 9 condylar cartilage were pooled together for homogenizing (n = 3), and the total RNA in control or experimental groups was extracted. For in vitro experiment, 5 × 10^5^ chondrocytes from each group were used for RNA extraction. Primers for targeted genes were designed as follow: CD163 (rat): forward, TCAGCGTCTCTGCTGTCACTCA, reverse, CGTTCATGCT CCAGCCGTTA; TNF-α (rat): forward, AACTCGAGTGACAAGCCCGTAG, reverse, GTACCACCAGTTGGTTGTCTTTGA; ACP-1(rat): forward, TCCTCAG TGCTGTGCCCAAG, reverse, ACAAATGCCTGATTTCCACCTACAA; MMP3 (rat): forward, TCTTTCACTCAGCCAATGCT, reverse, GGGAGGTCCATAG AGGGATT; MMP9 (rat): forward, AGCCGGGAACGTATCTGGA, reverse, TGGAA ACTCACACGCCAGAAG; Integrinα4 (rat): forward, CAGGCCATCCGTCTTGG AA, reverse, CGATGGTGAAATGCCGTTTG; Integrinβ1(rat): forward, TGCACA GATCCCAAGTTCCAAG, reverse, TGAAGGCTCTGCACTGAACACA; GAPDH (rat): forward, GGCACAGTCAAGGCTGAGAATG, reverse, ATGGTGGTGA AGACGCCAGTA; CD163 (human): forward, GGCTCAATGAAGTGAAGTGCA AAG, reverse, CCAAGGATCCCGACTGCAA; TNF-α (human): forward, GTGACA AGCCTGTAGCCCATGTT, reverse, TTATCTCTCAGCTCCACGCCATT; GAPDH (human): forward, GCACCGTCAAGGCTGAGAAC, reverse, TGGTGAAGACGCC AGTGGA. All genes were analyzed using the Applied Biosystems 7500 Real Time PCR machine. The amount of target cDNA, relative to GAPDH, was calculated using the formula 2^−ΔΔCt^. The results were calculated as the relative quantification of the target gene compared to the 4-wk control group or the amputated control cartilage, which was set at 1^1^.

**Western blotting**

In each group, every 3 out of 9 condyles cartilage were pooled together for homogenizing (n = 3), and the total protein in control or experimental groups was extracted as we previously described^3^. Briefly, total proteins from each group (40 μg) were fractionated by SDS-PAGE and transferred onto a nitrocellulose membrane. The nitrocellulose sheet was blocked with 5% nonfat milk and incubate with above anti-CD163 (1:200), anti-TNF-α (1:500). The blots were developed using a horseradish peroxidase–conjugated secondary antibody (1:5000, ZhongShan Goldenbridge Biotechnology, China) and enhanced chemiluminescence detection.

**Magnetic sorting CD163 positive cell**

Monosized magnetic polystyrene beads (25μl/1×10^7^cells) pre-coated with human anti-mouse IgG (Dynal 115.31D, Invitrogen, San Diego, CA) were washed once with 1ml chilled PBS containing 1% FCS (PBS-FCS) by placing the tube in the field of an MPC-1 magnet (Dynal) followed by removal of the buffer by suction. Beads were then resuspended in 25 μl of PBS-FCS and kept on ice. The mouse-anti-rat CD163 primary antibody (1 μg/10^6^ cells) was added into the cell suspension and mixed. After incubated in 4°C for 10 min, the cells were washed with 200 μl PBS-FCS and resuspended at the density of 1×10^7^cells/ml. Antibody-coated beads and cells were mixed and incubated for 20 min at 4°C with gentle tilting and rotation. The tube was placed in the magnet for 2 min, and then the supernatant containing CD163 negative cells was collected. Cell-bead clusters as well as free beads were retained on the wall of the tube while unbound cells were carefully removed by suction and transferred to a new tube. After incubation with the releasing buffer for 15 min at room temperature, the supernatant containing CD163 positive cell was collected by placing the tube in the magnet.

**Transwell migration assays**

In vitro migration assay was performed by using 24-well transwell units (Millipore, Merck KGaA, Darmstadt, Germany) with polycarbonate filters (pore size 8μm) coated both sides with fibronectin (3ng/ml, Sigma, USA).^6^ Isolated chondrocytes (4×10^4^) in 200 μl of DMEM with 10% FBS for each group were allowed to attach the upper part of the transwell unit for 48h at 37℃ in a fully humidified atmosphere with 5% CO_2_. After the pre-incubation, the medium of the upper part was replaced with 200 μl DMEM containing 1% FBS. The lower part of the transwell unit was filled with 500 μl DMEM (1% FBS), 500 μl DMEM (1% FBS) +5 ng TNF-α, and 500 μl DMEM (1% FBS) + 5 ng TNF for 6 h and sequentially 5ug TNF-α antibody for another 6h. After incubation for 12h under the same condition, non-migrated cells on the upper part of the membrane were removed with a cotton swab. Migrated cells on the bottom surface of the membrane were stained with mouse-anti-rat CD163 antibody followed the immunohistochemical protocol described above. Five fields at 200 × magnification were selected at random, and the CD163 positive cells and total cells in each image counted. Experiments were performed in triplicate.

**References**

1. Jiao K, Niu LN, Wang MQ, Dai J, Yu SB, Liu XD, et al. Subchondral bone loss following orthodontically induced cartilage degradation in the mandibular condyles of rats. Bone 2011;48(2):362-71.

2. Jiao K, Wang MQ, Niu LN, Dai J, Yu SB, Liu XD, et al. Death and proliferation of chondrocytes in the degraded mandibular condylar cartilage of rats induced by experimentally created disordered occlusion. Apoptosis 2009;14(1):22-30.

3. Yu SB, Wang MQ, Li YQ, Lv X, Jiang Y, Dong GY, et al. The effects of age and sex on the expression of oestrogen and its receptors in rat mandibular condylar cartilages. Arch Oral Biol 2009;54(5):479-85.
